# Morbid Elongation of Bones

**Published:** 1869-11

**Authors:** 


### Morbid Elongation of Bones.

Professor Langenbeck has recently read a paper before the Berlin Medical Society on the subject of "Morbid Increase of the Length of the Long Bones." In this paper he has called attention to the fact that the long bones, when subject to irritation during the growing period of life, are apt to increase in length and thickness. * * From his observations he draws three conclusions, viz.:

1.—Morbid causes which produce irritation, and hyperæmia of the bony tissue, have as a result, as long as the bone-growing period lasts, an increase in length, as well as in thickness of the bone.

2.—The increase of length concerns principally the diseased bone, but it can also be observed in a healthy bone of the same extremity.

3.—The bone lengthened through the increase of growth, retains its dimensions through life. An after shortening through resorption does not take place, even although the original cause—viz., the bone disease—should long since have ceased to exist.

He then makes the proposition—if it be not possible to artificially regulate the growth of bone and through that to hinder or accelerate it. With this view he made an experiment on a dog about eight weeks old, by inserting ivory pegs into the femur and tibia of the left side. About four months later the dog was killed, and on comparing the experimented bones with those on the opposite side, he found that, "the femur showed no alteration in shape, but the joint surfaces of both hip and knee joints were slightly smaller, the diaphysis slightly thickened and uneven . . . The tibia, in the diaphysis of which two ivory pegs had been inserted, showed these changes somewhat more marked. . . . On measurement, the femur and tibia both showed an increase of five millimetres in length, making in the whole limb an increase of ten millimetres." It appears from this that both bones presented elongation and thickening of the diaphysis; but the epiphysis had become somewhat smaller. Here, also, the fibula was lengthened to a corresponding extent as the tibia, though that could only have been caused by the extension exerted on it by the growing tibia; and, what is more remarkable, it had obtained this without losing its connection with the tibia, as took place in a case described by Parise.—*Berlin Correspondence of Lancet* and *Medical Gazette.*

